# Treatment Effect of Phototherapy with Low-Level Energy in Patients with Allergic Rhinitis: A Single-Arm Observational Study

**DOI:** 10.3390/medicina59020226

**Published:** 2023-01-26

**Authors:** Ju Wan Kang, Joo Ae Lim, Han Cheol Lee, Ju Ha Park, Seung Hwan Han

**Affiliations:** 1 Department of Otorhinolaryngology, Gangnam Severance Hospital, Yonsei University College of Medicine, Seoul 06237, Republic of Korea; 2 Department of Medical Device Engineering and Management, Gangnam Severance Hospital, Yonsei University College of Medicine, Seoul 06237, Republic of Korea

**Keywords:** allergic rhinitis, Rhinoconjuntivitis Quality of Life Questionnaire, visual analog scale, phototherapy

## Abstract

Allergic rhinitis is one of the most common health challenges and has a chronic and repetitive course that requires symptomatic treatment. We aimed to investigate the effect of phototherapy on allergic rhinitis and how long it takes to demonstrate treatment effect. Twenty-one patients who were diagnosed with allergic rhinitis using the skin prick test were enrolled. Red light (660 nm) and infrared light (940 nm) with a low power energy of 5 mW were used three times a day at intervals of at least 5 h. The Rhinoconjuntivitis Quality of Life Questionnaire (RQLQ) and a visual analog scale (VAS) were used to measure the changes in symptoms. The median RQLQ and VAS scores before treatment were 62 (49–81.5) and 3 (2–5) points, respectively. The RQLQ score improved significantly at two and four weeks after treatment (52 [39–62.5]) and 46.0 [30.5–57.0], respectively). The VAS scores also improved significantly at two and four weeks after treatment. Nasal obstruction and rhinorrhea improved significantly at one week after the procedure. Low-power (5 mW) light irradiation (660 nm red light and 940 nm infrared) was effective in improving the symptoms of allergic rhinitis. In addition, symptom improvement became clear approximately a week after use. Further studies are required to reach a definitive conclusion.

## 1. Introduction

Allergic rhinitis is one of the most common health challenges, and other symptoms, such as fatigue, headache, and red eyes, may cause discomfort in addition to typical symptoms, such as rhinorrhea, nasal obstruction, sneezing, and itching [1]. Although these symptoms are not life threatening, they have a significant impact on an individual’s daily activities and quality of life and pose a large socioeconomic burden [2].

Allergic rhinitis is an inflammatory reaction of the nasal mucosa mediated by immunoglobulin E (IgE). Cells, such as eosinophils, mast cells, and basophils, play a role in the release of inflammatory substances, such as histamine, cytokines, and eosinophilic cationic protein [1,3]. Allergic rhinitis is a disease with repeated and continuous symptoms, and, depending on the severity of the symptoms or the response to treatment, various treatments, such as avoidance of allergens, medications, intranasal sprays, and immunotherapies, have been used. Antihistamines, antileukotrienes, and intranasal corticosteroids are effective in controlling the symptoms of allergic rhinitis. However, some patients show reluctance to long-term medication use or do not show an effect after a medical treatment [4].

Ultraviolet and visible lights induce apoptosis of T lymphocytes, decrease the number and function of dendritic cells, and increase immunomodulatory cytokines, such as interleukin-10 (IL-10) and transforming growth factor-b (TGF-b) [5,6]. Phototherapy using ultraviolet and visible lights is being used to treat various inflammatory skin diseases with strong immunosuppressive effects [5,6]. Therefore, studies on new treatment methods for these patients are ongoing. There have been some reports on the effectiveness of phototherapy in patients with allergic rhinitis and chronic sinusitis [1,3,7,8,9,10,11,12,13,14]. In the case of allergic rhinitis, a complete treatment mechanism is not known; however, it is known to improve symptoms of allergic rhinitis by irradiating light into the nasal cavity of patients with allergic rhinitis to increase the metabolism and blood flow in the rhinitis mucous membrane and reduce inflammation and oxidative stress. It is well known that eosinophils play an important role in allergic inflammatory reactions, and, through a nasal smear, eosinophils can help in the diagnosis or evaluation of allergic rhinitis [4]. Koreck et al. reported that eosinophils decrease through nasal lavage after phototherapy [1]. However, only a few studies have reported the effectiveness of light therapy devices in patients with allergic rhinitis. 

Therefore, this study aimed to evaluate the effect of low-power irradiation on the intensity of allergic rhinitis symptoms by applying 660 nm visible light and 940 nm near-infrared light from light-emitting diode (LED) sources in patients diagnosed with allergic rhinitis. Most previous studies have compared symptoms at two or four weeks after treatment. Therefore, we also studied how symptoms changed according to the duration of use and investigated the change in eosinophils through nasal smear after four weeks of treatment. 

## 2. Materials and Methods

### 2.1. Population

We performed a single-arm observational trial to investigate the effect of intranasal phototherapy using 660 nm red light and 940 nm near-infrared light from LED light sources. Patients (aged between 18 and 65 years) who presented with symptoms of allergic rhinitis for at least 2 years were offered to participate in the study. We classified the patients as intermittent (<4 days/week or <4 weeks/episode) and persistent (≥4 days/week and ≥4 weeks/episode) according to the ARIA guidelines. If it interferes with sleep, study, work, or daily life, it is classified as severe, and if not, it is classified as mild.

Allergy was confirmed using a skin prick test for 28 allergens. Patients who showed positive results for at least one allergen were included in this study. Patients with negative results in the skin prick test, those who were undergoing allergic immunotherapy, those who had asthma treatment, and those who were taking systemic steroids or immunosuppressants were excluded. Patients with findings that were suggestive of sinusitis, such as polyps or purulent rhinorrhea on nasal endoscopy, were also excluded. If the symptoms were severe, antihistamine was allowed to be taken to alleviate the symptoms. Detailed information regarding this study was provided, and informed consent was obtained from all enrolled participants. The Institutional Review Board of Gangnam Severance Hospital, Yonsei University College of Medicine, approved this study in accordance with the Declaration of Helsinki (approval number: 3-2022-0050).

### 2.2. Equipment and Methods for Use

A phototherapy device emitting 660 nm of visible light and 940 nm of infrared light with a low power energy of 5 mW (LUZNOSE CARE, VIVOZON Healthcare, Yongin, Korea) was used in this study. The device was operated after the probe was inserted into both nostrils simultaneously. Phototherapy was administered three times a day for four weeks at intervals of more than five hours so that the treatment was not too concentrated, as described in previous studies [10,11].

### 2.3. Assessment of Symptoms

Before the start of treatment, and at 2 and 4 weeks after treatment, the participants visited the clinic and filled out a symptom questionnaire, including the Rhinoconjuntivitis Quality of Life Questionnaire (RQLQ) and a visual analog scale (VAS). Moreover, the VAS on a 10-point scale was self-recorded at home every day for symptoms of rhinorrhea, nasal congestion, sneezing, and itching. 

### 2.4. Nasal Smear 

A nasal smear was performed twice: before treatment and at 4 weeks after treatment. Using a 0° endoscope, a sterile cotton swab was used to obtain the samples for the nasal smears along the inferior turbinate. The samples were smeared on a glass slide, and the nasal smears were examined using a light microscopy after staining with hematoxylin and eosin. The patients were divided into the following four stages according to the ratio of eosinophils: no eosinophilia (<5% of eosinophils), slight eosinophilia (≥5 and <10% of eosinophils), moderate eosinophilia (≥10% and <50% eosinophils), and severe eosinophilia (>50% of eosinophils) [4].

### 2.5. Statistical Analysis

Statistical analysis was performed using the SPSS Software (SPSS Inc. Released 2009. PASW Statistics for Windows, version 26.0. Chicago: SPSS Inc., Chicago, IL, USA) and R (R 4.13). The normal distribution of the data was confirmed using the Kolmogorov–Smirnov tests. If the normal distribution was confirmed, a paired t-test was performed. If the normal distribution was not followed, the Wilcoxon signed-rank test was used for data that did not have a normal distribution. All statistical tests were two-tailed, with significance levels (*p* ≤ 0.05).

## 3. Results

**Figure 1:**
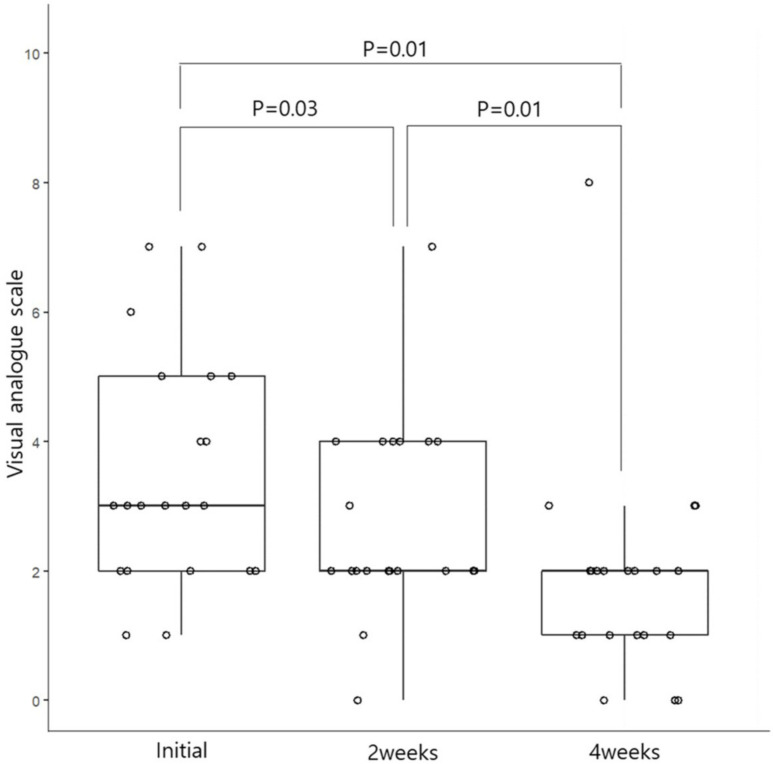
Changes in the visual analog scale score at 2 and 4 weeks before and after phototherapy among patients with allergic rhinitis.

**Figure 2:**
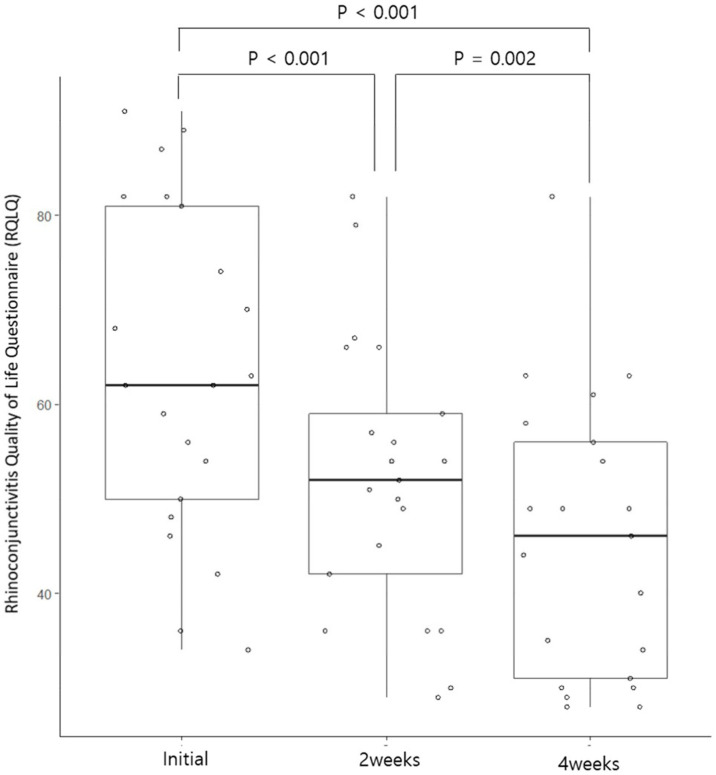
Changes in the Rhinoconjuntivitis Quality of Life Questionnaire score at 2 and 4 weeks before and after phototherapy among patients with allergic rhinitis.

A total of 21 patients participated in the study, comprising six men and fifteen women. The median age of the patients was 41.9 (±8.7, 25–57) years. None of our patients showed severe symptoms according to the ARIA classification that interfered with study, work, and sleep. In addition, all patients’ symptoms corresponded to mild, persistent allergic rhinitis. In addition, the VAS score was less than five points among the 21 patients, except for three patients. Antihistamines were allowed to be taken if the symptoms were severe, but no patients took the drug during the treatment period. When looking at the nasal symptom scale of the RQLQ survey, it was reported that symptoms improved in 29% of nasal congestion, 48% of runny nose, 52% of sneezing, and 57% of itching after two weeks of treatment, and 57% of symptoms improved, respectively. In the case of the VAS, it was confirmed that the score decreased from 52% after two weeks to 86% after four weeks. The median RQLQ and VAS scores before treatment were 62 (49–81.5) and 3 (2–5) points, respectively. After two weeks of treatment, the median value was lowered to 52 (39–62.5) and 2 (2–4) points, respectively, and the symptom score was significantly lower after two weeks of treatment when compared using the Wilcoxon signed-rank test (Figure 1 and Figure 2).

In the case of symptom score at four weeks after treatment, the median value of the RQLQ was 46.0 (30.5–57.0), showing a statistically significant improvement compared to before treatment and two weeks after treatment. In the case of the VAS, the median value was two (1–2) points after four weeks of treatment, indicating statistically significant symptom improvement compared to before treatment and after two weeks of treatment (Figure 1 and Figure 2).

**Figure 3:**
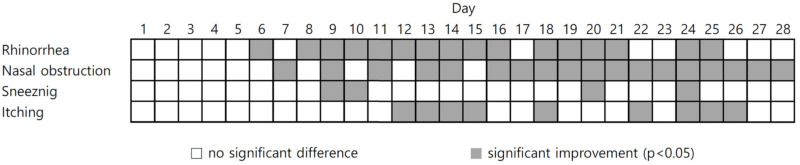
Daily changes in symptoms after phototherapy in patients with allergic rhinitis.

When comparing the effect on daily symptoms such as nasal obstruction, runny nose, sneezing, and itching before and after treatment, statistically significant improvement effects for runny nose and nasal congestion were con-firmed approximately 7 days after treatment. There were many daily changes in itching and sneezing (Figure 3). Therefore, the significant difference in the VAS score shown in Figure 1 might be due to changes in daily symptoms, such as nasal observation or rhinorrhea.

**Figure 4:**
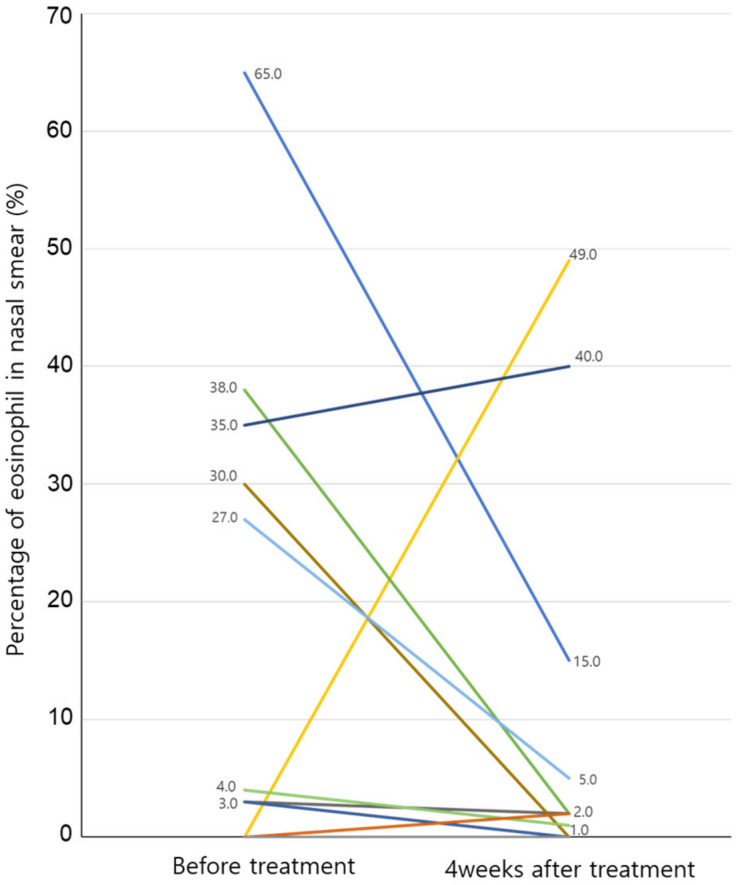
Changes in eosinophils before and after phototherapy with nasal smear. Eosinophilic percentage in the nasal smear is 0% in both tests before treatment and in 10 patients at 4 weeks after treatment. There are two patients who decrease from 3% before treatment to 0% after 4 weeks of treatment, and the results of the remaining patients can be seen in the figure.

The nasal smear test before treatment showed 15 participants with no eosinophilia, 4 participants with moderate eosinophilia, and 1 participant with severe eosinophilia. After four weeks of treatment, 1 in 15 participants with no eosinophilia showed moderate eosinophilia, and the rest showed no change. One participant with severe eosinophilia showed improvement to moderate eosinophilia. Of the four participants with moderate eosinophilia, two showed no eosinophilia, one demonstrated mild eosinophilia, and one showed moderate eosinophilia without change. The changes in eosinophils before and after treatment for the patients are presented in Figure 4.

## 4. Discussion

The results showed that low-power (5 mW) phototherapy with 660 nm red light and 940 nm infrared light was effective in improving the symptoms of allergic rhinitis. Symptom improvement was statistically significant for the RQLQ and VAS scores at two and four weeks after treatment compared to the RQLQ and VAS scores before treatment. When investigating the degree of symptom improvement after using the device for each symptom, symptoms tended to improve significantly in the case of runny nose and nasal congestion at seven days after the start of treatment. No significant adverse reactions occurred during the treatment period, but a temporary adverse reaction indicated that one participant had a dry nose for approximately two days, and there was temporary itching of the nose.

There were no severe adverse reactions to phototherapy in previous reports [1,3,7,8,9,10,11]. However, in the case of devices using ultraviolet (UV), the dryness of the nose was a common complaint; however, when only visual light was used, this abnormal reaction was low, and the same result was shown in our study [8]. Considering that some patients showed nasal dryness, the effect of phototherapy on mucociliary function may also be questioned. Previous study showed that there was no difference in mucociliary function between patients undergoing phototherapy and a control group when mucociliary function was measured using a saccharin test [12].

The mechanism of phototherapy in allergic rhinitis has not yet been identified. However, total IgE and IL-4 levels were significantly reduced when the infrared-wavelength visible light used in this study was applied to an allergic rhinitis mouse model [15]. Other studies have also found that IL-4, IL-17, and IgE levels are inhibited by infrared-wavelength visible light in mice with allergic rhinitis, and eosinophil infiltration into the nasal mucosa was also significantly decreased [16]. Koreck et al. showed that eosinophils, eosinophilic protein, and Il-5 from nasal secretion collected using nasal lavage were significantly decreased after phototherapy. However, eosinophils increased despite symptom improvement [17], and eosinophil cationic protein did not show differences with phototherapy [10]. In this study, five out of twenty-one participants showed a moderate or higher increase in eosinophils, four patients showed a decrease in eosinophils after four weeks of treatment, and there was no change in one patient. Although statistical significance could not be found due to the small number of patients whose eosinophils were measured, it was found that eosinophils tended to decrease after phototherapy. Further studies using nasal lavage or smear are necessary to make concrete conclusions.

In addition to rhinitis, there has been a study on the effectiveness of phototherapy for chronic sinusitis. Krespi et al. showed that near-infrared laser illumination had a beneficial effect on patients with chronic sinusitis, and the treatment effect lasted for approximately two months [13]. However, one study reported that phototherapy using mixed visible and ultraviolet lights was not effective for the treatment of chronic sinusitis without polyps [12].

This study has several limitations. First, this was an observational study in a single group. We could not analyze the effect of symptom improvement when compared to a control group. Symptom improvement with phototherapy has been confirmed in previous studies. However, previous studies have mainly analyzed the therapeutic effects at two or four weeks. We found that symptoms improved significantly after approximately seven days of phototherapy by checking the daily symptom scores. Secondly, previous studies have reported that wearing face masks can relieve the symptoms of allergic rhinitis. This study was conducted during the COVID-19 pandemic, and it was mandatory to wear a mask indoors and outdoors in South Korea. Wearing masks due to COVID-19 might have affected the participants’ symptoms [18,19]. However, phototherapy significantly improves symptoms even when symptoms are not severe, which is thought to be a significant result in allergic rhinitis that shows progress toward becoming a chronic disease. Third, only subjective improvement using phototherapy was observed. It would be better if objective tests, such as acoustic rhinometry and acoustic rhinomanometry, were conducted. The two previous studies used peak nasal flow and showed different results. In one study, peak nasal flow also increased with improved symptoms [11], but in another study, there was no difference in peak nasal flow [10]. We conducted nasal smears to assess the severity of inflammation. Moreover, eosinophilia tended to decrease slightly. However, the number of participants to compare the change in the eosinophil count before and after phototherapy was small. Finally, there was an insufficient number of participants and a lack of long-term results to reach a concrete conclusion.

There are no research results that can be used to make conclusions on the type or energy of light rays used in light therapy and the frequency or duration of use. Previous studies have used various types of light, including a mixture of UVA/UVB and visual light [1,3,7,9,11,14], infrared and visual light [10], and visible light [8,9,17]. (Table 1).

To the best of our knowledge, no study has compared the effects of different light sources to date. However, considering the progress of chronic allergic rhinitis, light therapy is thought to be an alternative for patients who do not show improvement in symptoms or who show resistance to drugs. In addition, further studies on the effect of treatment for longer-term use or the change in symptoms after discontinuation of treatment should be conducted when we consider the chronic disease progress of allergic rhinitis.

## 5. Conclusions

Low-power (5 mW) light irradiation (660 nm red light and 940 nm infrared) was effective in improving the symptoms of allergic rhinitis. In addition, symptom improvement became clear approximately a week after use. Phototherapy could be considered as an alternative treatment for patients with allergic rhinitis. 

## Figures and Tables

**Table 1:** Direction and wavelength of phototherapy.

Light Type	Wave Length	Directions	References
UV-A, UV-B, and VIS	310–620 nm	3 times/week for 3 weeks	Koreck et al. [1]
VIS, and Infrared	652 nm, 940 nm	3 times/day for 2 weeks	Emberlin et al. [10]
UV-A, UV-B, and VIS	310–620 nm	3 times/week for 2 weeks	Cingi et al. [7]
UV-A, UV-B, and VIS	310–620 nm	2 times/week for 3 weeks	Tatar et al. [14]
VIS	650 nm	2 times/day for 4 weeks	Lee et al. [8]
UV-A, UV-B, and VIS	310–620 nm	3 times/week for 2 weeks	Alyasin et al. [3]
UV-A, UV-B, and VIS	310–620 nm	3 times/week for 1 week and 2 times/weeks for next 5 weeks	Bella et al. [11]
VIS, and Infrared	660 nm, 940 nm	2 times/day for 5 weeks	Kennedy et al. [9]
VIS, and Infrared	660 nm, 940 nm	2 times/day for 2 weeks and 1 time/day for next 2 weeks	Koycu et al. [17]

Abbreviation: UV-A; ultraviolet-A, UV-B; ultraviolet-B, and VIS: visible light.

## Data Availability

The authors confirm that the data supporting the findings of this study are available within the article. Other raw data are not publicly available as they contain information that could compromise the privacy of research participants and regulation of data sharing in the Republic of Korea.
